# Exogenously overexpressed intronic long noncoding RNAs activate host gene expression by affecting histone modification in Arabidopsis

**DOI:** 10.1038/s41598-020-59697-7

**Published:** 2020-02-20

**Authors:** Zhang-Wei Liu, Nan Zhao, Yin-Na Su, Shan-Shan Chen, Xin-Jian He

**Affiliations:** 1 National Institute of Biological Sciences, Beijing, 102206 China; 2 Tsinghua Institute of Multidisciplinary Biomedical Research, Tsinghua University, 10084 Beijing, China

**Keywords:** Plant biotechnology, Plant development, Plant genetics

## Abstract

Involvement of long non-coding RNAs (lncRNAs) in the regulation of gene expression in *cis* has been well studied in eukaryotes but relatively little is known whether and how lncRNAs affect gene expression in *tans*. In *Arabidopsis thaliana*, *COLDAIR*, a previously reported lncRNA, is produced from the first intron of *FLOWERING LOCUS C* (*FLC*), which encodes a repressor of flowering time. Our results indicated that the exogenously overexpressed *COLDAIR* enhances the expression of *FLC* in *trans*, resulting in a late-flowering phenotype. In *35S-COLDAIR* lines, the enhanced expression of *FLC* is correlated with the down-regulation of the repressive histone mark H3K27me3 and with the up-regulation of the active histone mark H3K4me3 at the *FLC* chromatin. Furthermore, we demonstrated that overexpression of intronic lncRNAs from several other H3K27me3-enriched MADS-box genes also activates the expression of their host genes. This study suggests that the involvement of overexpressed intronic lncRNAs in gene activation may be conserved in H3K27me3-enriched genes in eukaryotes.

## Introduction

Long noncoding RNAs (lncRNAs) which constitute a large portion of the transcriptome are involved in diverse biological processes in eukaryotes^1,2^. It is well known that lncRNAs can regulate gene expression by interacting with chromatin modifiers^3,4^. *Xist* (*X inactive specific transcript*), an lncRNA transcribed from the inactive X chromosome (Xi), leads to the recruitment of PRC2 (Polycomb Repressive Complex 2) which deposits the repressive H3K27me3 modification across the Xi and orchestrates X chromosome inactivation (XCI)^5,6^. *HOTAIR*, an lncRNA derived from the antisense strand of the *HOXC* locus, interacts with PRC2 and is required for the association of PRC2 with chromatin and H3K27me3 at the *HOXD* locus^7^. Unlike the lncRNAs which promote H3K27me3, *HOTTIP*, a lncRNA transcribed from the 5′ end of the *HOXA* locus, targets the histone H3K4 methyltransferase complex COMPASS to *HOXA*, driving histone H3K4me3 and gene activation^8^. In plants, thousands of lncRNAs were identified by high-throughput sequencing and were demonstrated to be involved in diverse biological processes^1,2,9,10^. Previously characterized lncRNAs were shown to be important for flowering time^11,12^, photomorphogenesis^13^, male sterility^14–16^, grain yield^17^, pathogen resistance^18^, and phosphate starvation^19^.

FLOWERING LOCUS C (FLC) is a MADS-box protein that acts as a key repressor of flowering in *Arabidopsis thaliana*^20^. The repressive histone modification H3K27me3 functions antagonistically with the active histone modifications H3K4me3 and H3K36me3 to regulate the transcription of *FLC*^21^. Polycomb Group (PcG) complex is responsible for H3K27me3 and thus mediates transcriptional repression of *FLC*^22,23^. CURLY LEAF (CLF) is a well-known histone H3K27 trimethyltransferase that functions as a catalytic subunit of the PcG complex^24–26^. The transcriptional activation of *FLC* requires Trithorax class H3K4 methyltransferases such as ARABIDOPSIS TRITHORAX-LIKE PROTEIN 1 (ATX1), which mediates the establishment of H3K4me3^27^. The H3K27me3 level at the *FLC* chromatin is reduced in the *clf* mutant and the reduction of H3K27me3 is accompanied by increased H3K4me3^23^, indicating that CLF may indirectly repress H3K4me3 at the *FLC* chromatin. Upon transition to flowering, the removal of H3K4me3 is accompanied by an increased level of H3K27me3, leading to reduced *FLC* expression^27^.

Epigenetic modification of the *FLC* chromatin involves cis-acting lncRNAs, including *COOLAIR*, *COLDAIR*, and *COLDWRAP*^11,12,28^. *COOLAIR* is a set of alternative spliced and polyadenylated antisense lncRNAs transcribed from the 3′ UTR of *FLC*^11^. *COOLAIR* mediates the replacement of H3K36 methylation with H3K27me3 but work independently of the PcG complex during the early stage of vernalization^29^. *COLDAIR*, an intronic lncRNA from the first intron of *FLC*, cooperates with the *FLC* promoter-derived lncRNA *COLDWRAP* to facilitate the establishment of H3K27me3 and to thereby repress *FLC* expression during the late stage of vernalization^12,28^. Considering that *COLDAIR* is a functional lncRNA generated from the intron of *FLC*, we wonder how *COLDAIR* regulates *FLC* and whether there other intronic RNAs involved in regulating the expression of their corresponding host genes.

Intronic lncRNAs have been extensively identified and demonstrated to be functional in regulating the expression of their corresponding host genes in eukaryotes^30^. Generated from spliced introns, circular intronic RNAs (ciRNAs) are abundant in the nucleus and promote the transcription of their host genes by associating with RNA polymerase II in human cells^31^. Stable intronic RNAs were also found to play an important role in enhancing the expression of their host genes in *Xenopus* and *Drosophila*^32,33^. Although the function of intronic RNAs in the regulation of transcription has been extensively studied, it remains largely unknown how these intronic RNAs regulate transcription through affecting chromatin modification.

In this study, we demonstrate that the exogenously overexpressed intronic noncoding RNA *COLDAIR* is sufficient to enhance *FLC* expression in multiple independent *COLDAIR* transgenic lines. The enhancement depends on the recruitment of the H3K4me3 methyltransferase ATX1 and the removal of the H3K27 trimethyltransferase CLF at the *FLC* chromatin. Furthermore, we demonstrate that overexpression of intronic lncRNAs derived from several other H3K27me3-enriched MAD-box genes is also sufficient to enhance the expression of their corresponding host genes. These results show that intronic lncRNAs derived from H3K27me3-enriched MADS-box genes can enhance the expression of their corresponding host genes by suppressing H3K27me3 and promoting H3K4me3. The study suggests that ectopically overexpressed intronic RNAs may regulate the expression of their host genes by affecting the occupancy of histone methyltransferases.

## Results

### Ectopically overexpressed *COLDAIR* enhances *FLC* expression *in vivo*

**Figure 1:**
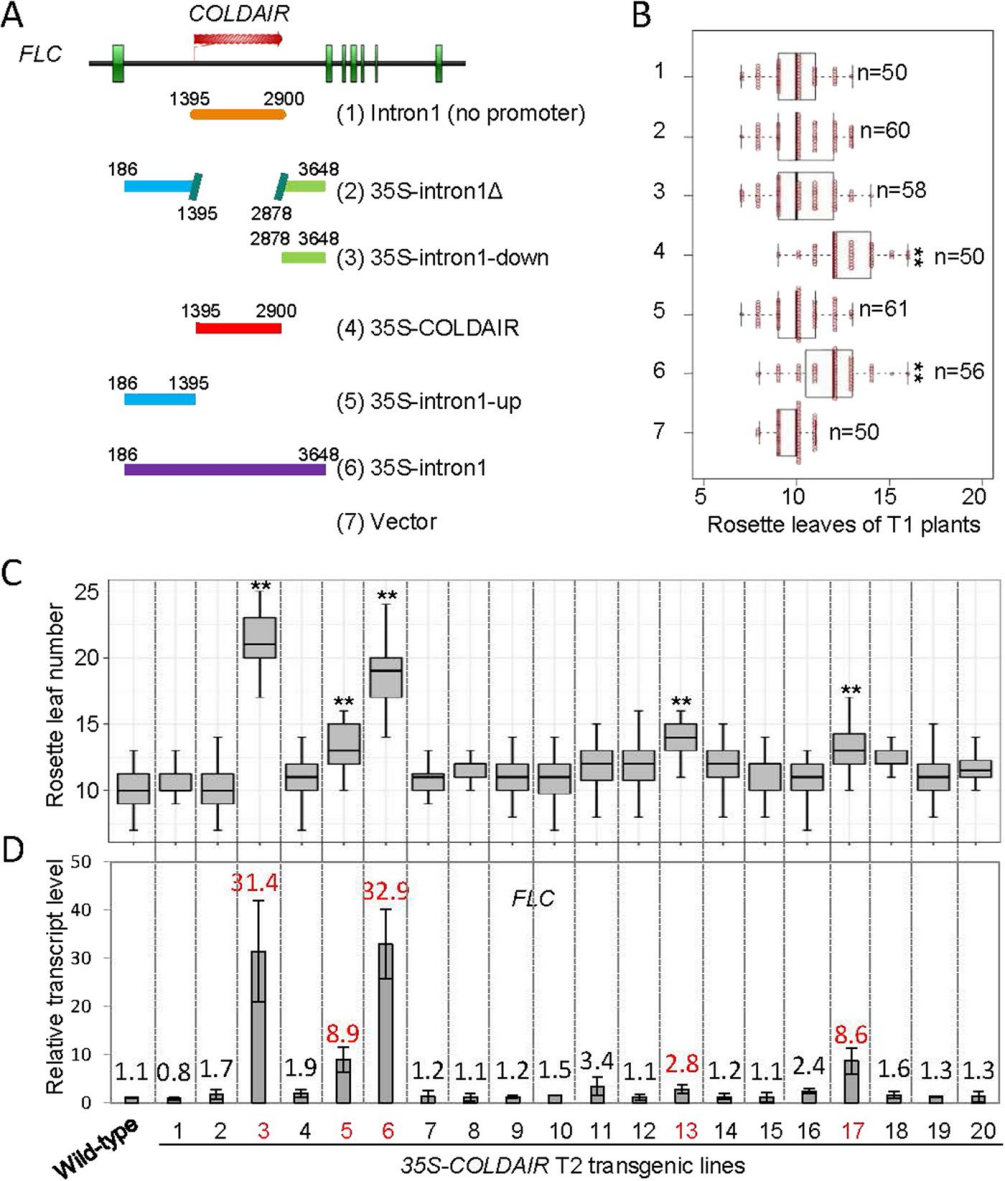
The exogenously overexpressed intronic noncoding RNA *COLDAIR* promotes the expression of its host gene *FLC*. **(A)** Diagrams indicating the truncated versions of *FLC* that were transformed into plants with or without the *CaMV 35S* promoter. **(B)** Box plots showing flowering times of randomly selected T1 transgenic plants. Flowering time was measured based on the number of rosette leaves, and “n” denotes the number of T1 transgenic plants. The number of rosette leaves for all tested plants is shown. Asterisks indicate that differences between wild-type plants and each set of transgenic plants were statistically significant (Student’s t test; **p < 0.001). **(C)** Flowering times of *35S-COLDAIR* T2 transgenic lines. Thirty-six T2 plants were scored for each line. Asterisks indicate that differences between wild-type plants and each of transgenic lines were statistically significant (Student’s t test; **p < 0.0001, *p < 0.001). **(D)** Transcript levels of *FLC* in 20 independent *35S-COLDAIR* T2 transgenic lines and in the wild type. A mix of at least 10 T2 transgenic plants were used for determination of the *FLC* transcript level in each transgenic line.

FLC is a MADS-box-containing transcriptional factor that functions as a critical flowering repressor in Arabidopsis^20^. The first intron of *FLC* is required for transcriptional regulation^22,34,35^. *COLDAIR*, an intronic lncRNA from the first intron of *FLC*, was known to promote H3K27me3 and thereby repress the transcription of *FLC*^12^, but how *COLDAIR* regulates H3K27me3 is largely unknown^36^. We developed a transgene system, in which the full-length or truncated sequences of the first *FLC* intron were overexpressed under the control of the *Cauliflower Mosaic Virus* (*CaMV*) *35S* promoter (Fig. 1A). FLC functions in a dosage-dependent manner to control flowering time in Arabidopsis^20,37^. In this transgene system, we found that the *35S-COLDAIR* T1 transgenic plants expressing the full-length *COLDAIR* showed significantly late flowering, whereas the transgenic plants harboring the *COLDAIR* sequence without the *35S* promoter did not exhibit significant changes in flowering time (Fig. 1A,B). To confirm the effect of the *COLDAIR* overexpression, we randomly selected 20 individual *35S-COLDAIR* T2 transgenic lines for determining *FLC* expression and flowering time. The results showed that 25% (5/20) of randomly selected *35S-COLDAIR* lines displayed late flowering, which is accompanied by the increased expression of *FLC* (Fig. 1C,D). The results suggest that the ectopic overexpression of *COLDAIR* can enhance the expression of *FLC in vivo*. We assessed the transcript levels of *FLC* and *COLDAIR* in different ecotypes of Arabidopsis by RT-qPCR. The result indicated that the transcript levels of *FLC* were different in the indicated ecotypes (Fig. S1). The different expression levels of *FLC* in these ecotypes are primarily due to nature variants of FRIGIDA, a transcriptional activator of *FLC*^38^. Moreover, the levels of *COLDAIR* are positively correlated with the *FLC* transcript levels in the indicated ecotypes (Fig. S1), suggesting that *COLDAIR* is unlikely to play a critical role in the repression of *FLC* expression.

### The ectopically overexpressed *COLDAIR* specifically targets *FLC*

A high level of *FLC* expression is characteristic of the vernalization-responsive winter annual ecotypes of Arabidopsis, which are different from the rapid-cycling early-flowering ecotypes^20,38^. Vernalization reduces *FLC* expression and eliminates the late-flowering phenotype in the winter annual ecotypes. To elucidate the mechanism underlying the late-flowering phenotype of the *35S-COLDAIR* lines, we determined whether the effect of the *COLDAIR* overexpression on flowering time is related to vernalization. Two representative late-flowering *35S-COLDAIR* lines (*35S-COLDAIR #3* and *#17*) with or without vernalization treatment were chosen for the analysis of flowering time. The results showed that the late-flowering phenotype of both transgenic lines was corrected by the vernalization treatment (Fig. S2), which supports the notion that the late-flowering phenotype of the *COLDAIR* transgenic lines is caused by increased expression of *FLC*.

**Figure 2:**
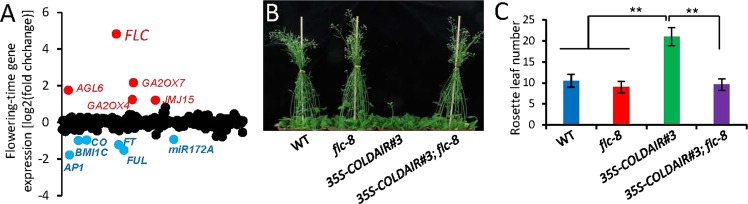
The overexpressed *COLDAIR* represses flowering by promoting the expression of *FLC*. **(A)** The effect of *35S-COLDAIR #3* on the expression of flower time-related genes as determined by RNA-seq. Each dot represents one flowering time-related gene. Significantly up- and down-regulated genes are shown in red and blue, respectively. **(B)** The late-flowering phenotype of *35S-COLDAIR #3* is eliminated in *flc-8* mutant plants under long-day conditions. The *flc-8* mutation was introduced into *35S-COLDAIR #3* by genetic crossing. **(C)** Flowering times of indicated plants measured by the number of rosette leaves. Asterisks indicate statistically significant differences (Student’s t test; **p < 0.01).

To investigate the effect of the *COLDAIR* overexpression at the whole-genome level, we performed RNA deep sequencing (RNA-seq) in order to compare the expression of all the flowering time related genes between the representative *35S-COLDAIR* line (*35S-COLDAIR #3*) and the wild type (Table S1). Our RNA-seq data showed that the expression of *FLC* was markedly increased in *35S-COLDAIR #3* (Fig. 2A; Data set 1). Concomitantly, the expression of the key flowering promoter gene *FLOWERING LOCUS T* (*FT*) and of the critical floral meristem identity gene *APETALA1* (*AP1*) was significantly reduced (Fig. 2A; Data set 1). Because *FLC* reduces the expression of *FT* and *AP1*^39,40^, reduced expression of *FT* and *AP1* is likely to be caused by the increased expression of *FLC*. To further verify whether a high level of *FLC* expression is required for the late-flowering phenotype, we crossed *35S-COLDAIR #3* with a loss-of function *flc* mutant (*flc-8*) to introduce the *flc* mutation into the *35S-COLDAIR* plants. The results showed that the late-flowering phenotype of *35S-COLDAIR #3* was completely suppressed by the *flc* mutation (Fig. 2B,C). These data demonstrate that the late-flowering phenotype of the *35S-COLDAIR* plants can be eliminated by the *flc* mutation, and indicate that the increased expression of *FLC* is responsible for the late-flowering phenotype of the *35S-COLDAIR* plants.

### The exogenous *COLDAIR* functions in the form of double-stranded RNAs *in vivo*

**Figure 3:**
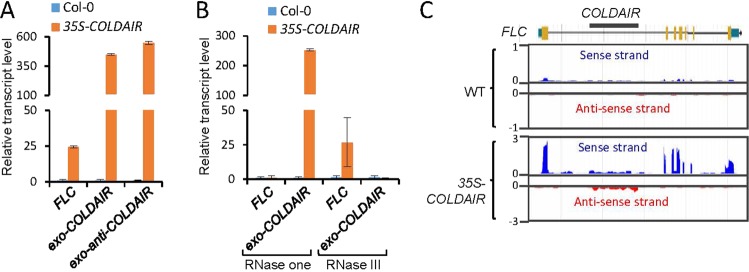
The exogenous transgenic *COLDAIR* produces double-stranded RNA *in vivo*. **(A)** RT-qPCR analysis of *FLC* mRNA and sense and antisense strands of exogenous *COLDAIR* in the wild-type Col-0 and in the *35S-COLDAIR* transgenic line. Error bars are SD of three replicates. **(B)** Analysis of *FLC* mRNA and exogenous *COLDAIR* with or without RNase treatment *in vitro*. RNase one (Promega, M4261) is an ssRNA specific ribonuclease, and RNase III (NEB, M0245) mediates the cleavage of double-stranded RNAs. **(C)** Snapshots of sense and antisense *FLC* transcripts in the wild type and the *35S-COLDAIR* transgenic plants. Sense and antisense transcripts are shown in blue and red, respectively.

To investigate how the exogenous *COLDAIR* transcripts affect the expression of *FLC* in the *35S-COLDAIR* plants, we performed RT-qPCR to detect the exogenous *COLDAIR* transcripts, and found that both sense- and antisense-strands of the exogenous *COLDAIR* RNA were significantly increased (Fig. 3A). To assess whether the exogenous *COLDAIR* is in the form of double-stranded RNAs (dsRNAs), total RNA was treated with RNase one, which specifically digests single-stranded RNAs (ssRNAs), or with RNase III, which digests only dsRNAs, followed by RT-qPCR^41^. The results showed that, without the RNase treatment, the levels of both the *FLC* mRNA and the exogenous *COLDAIR* were significantly higher in the *35S-COLDAIR* plants than in the wild type (Fig. 3B). After treated with RNase one, the high *FLC* mRNA level in the *35S-COLDAIR* lines was markedly reduced, whereas the exogenous *COLDAIR* transcript level was only slightly reduced (Fig. 3B). After treated with RNase III, the *FLC* mRNA level remained significantly higher in the *35S-COLDAIR* plants than in the wild-type plants, whereas the exogenous *COLDAIR* transcripts were almost completely restored to the wild-type level (Fig. 3B). These results suggest that, while the *FLC* mRNA occurs as ssRNAs, the *35S-COLDAIR* produces dsRNAs.

We performed strand-specific transcriptome analysis in the wild type and the *35S-COLDAIR* plants to further determine whether the *COLDAIR* transcripts can form dsRNAs. The data showed that the *FLC* mRNA level was significantly increased in the *35S-COLDAIR* plants (Fig. 3C), which is consistent with the RNA-seq data indicated above (Fig. 2C). Both sense and antisense RNAs of *COLDAIR* were significantly higher in the *35S-COLDAIR* plants than in the wild type (Fig. 3C). These results therefore suggest that the exogenously expressed *COLDAIR* results in the production of dsRNAs.

### The exogenously expressed *COLDAIR* RNA may directly promote *FLC* expression without producing small RNAs or small peptides

**Figure 4:**
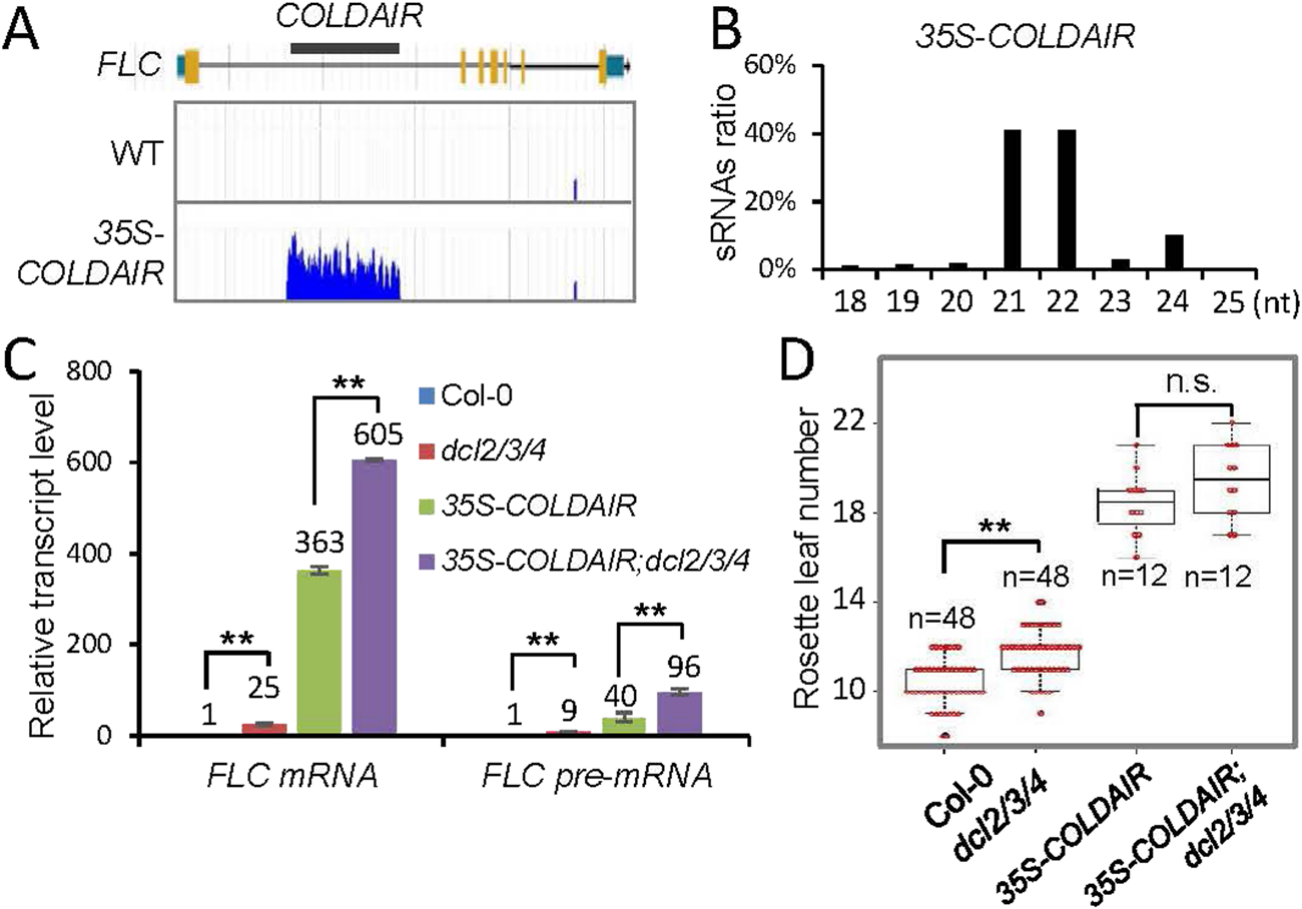
The effect of *COLDAIR* overexpression on activation of *FLC* is independent of the production of small RNAs. **(A)** Snapshots of small RNAs derived from *COLDAIR* in the wild type and the *35S-COLDAIR* #3 transgenic line. **(B)** Percentage of different sizes of small RNAs derived from the *COLDAIR* locus in the *35S-COLDAIR* #3 transgenic line as determined by small RNA deep sequencing. **(C)** The effect of *dcl2/3/4* on levels of *FLC* mRNA and *COLDAIR* transcripts in the wild type and the *35S-COLDAIR* #3 transgenic line as determined by RT-qPCR. Error bars are SD of three replicates. Asterisks indicate the significance of difference (Student’s t test; **p < 0.01). **(D)** The effect of *dcl2/3/4* on flowering time in the wild type and the *35S-COLDAIR* #3 transgenic line. Numbers of rosette leaves on the indicated plants are shown by box plots. “n” denotes the number of plants scored for each genotype. Asterisks indicate the significance of difference (Student’s t test; **p < 0.01). “n.s.”, not statistically significant.

Small RNAs derived from dsRNAs can not only suppress but also activate transcription^42^. To investigate whether small RNAs are generated from the *COLDAIR* dsRNA, we carried out small RNA deep sequencing in wild-type and *35S-COLDAIR* plants. The data showed that small RNAs from the *COLDAIR* sequence were enriched in the *35S-COLDAIR* plants but not in the wild-type plants (Fig. 4A). The sizes of these small RNAs were predominantly 21 nt, 22 nt, and to a lesser extent 24 nt (Fig. 4B). In Arabidopsis, there are four DCLs (Dicer-like proteins) responsible for generating distinct small RNAs. Whereas DCL1 is responsible for the cleavage of ssRNAs into 21-nt miRNAs, the other three DCLs are responsible for generating small RNAs from dsRNA precursors^43,44^. DCL2 plays a role in the formation of 22-nt natural antisense or viral siRNAs; DCL3 is involved in the biogenesis of 24-nt heterochromatic siRNAs; and DCL4 produces 21-nt siRNAs from inverted repeated (IR) genes and ta-siRNAs^45^.

Given that the exogenous *COLDAIR* produces small RNAs, we determined whether DCLs contribute to the regulation of *FLC* expression via generating the small RNAs. We introduced the *35S-COLDAIR* transgene into the *dcl2/3/4* triple mutant by genetic crossing, and then compared the effects of the exogenous *COLDAIR* on *FLC* expression between the wild-type and *dcl2/3/4* mutant backgrounds. Our RT-qPCR results showed that, while *FLC* expression was enhanced by both the overexpressed *COLDAIR* and the *dcl2/3/4* mutation, *FLC* expression was significantly enhanced by the *dcl2/3/4* mutation in the *35S-COLDAIR* line (Fig. 4C). The late-flowering phenotype of the *35S-COLDAIR* line was slightly enhanced by the *dcl2/3/4* mutation but the enhancement was not significant as determined by a statistical analysis (Fig. 4D). It is possible that the increase in *FLC* expression caused by the *dcl2/3/4* mutation is not sufficient to further delay flowering in the *35S-COLDAIR* background. These results indicated that, whether or not *COLDAIR* is overexpressed, DCL2/3/4 suppress rather than promote *FLC* expression, suggesting that the full-length exogenous *COLDAIR* RNAs, but not the small RNA byproducts, are responsible for the promotion of *FLC* expression.

Small peptides from lncRNAs were previously found to be involved in the regulation of gene expression and development^46,47^. Thus, the exogenous *COLDAIR* may enhance *FLC* expression by encoding small peptides. According to the putative open reading frames (ORFs) in *COLDAIR*, we truncated the *COLDAIR* sequences into three parts, which may translate different versions of peptides (Fig. S3A). Although the three truncated *COLDAIR* sequences were overexpressed under the control of the *35 S* promoter in their corresponding transgenic plants, they failed to significantly affect *FLC* expression or flowering time (Fig. S3B,C). The results suggest that the exogenous *COLDAIR* transcripts do not promote *FLC* expression by expressing small peptides. Thus, the full-length exogenous *COLDAIR* transcripts may directly enhance *FLC* expression *in vivo*.

### The exogenously overexpressed *COLDAIR* reduces H3K27me3 and increases H3K4me3

**Figure 5:**
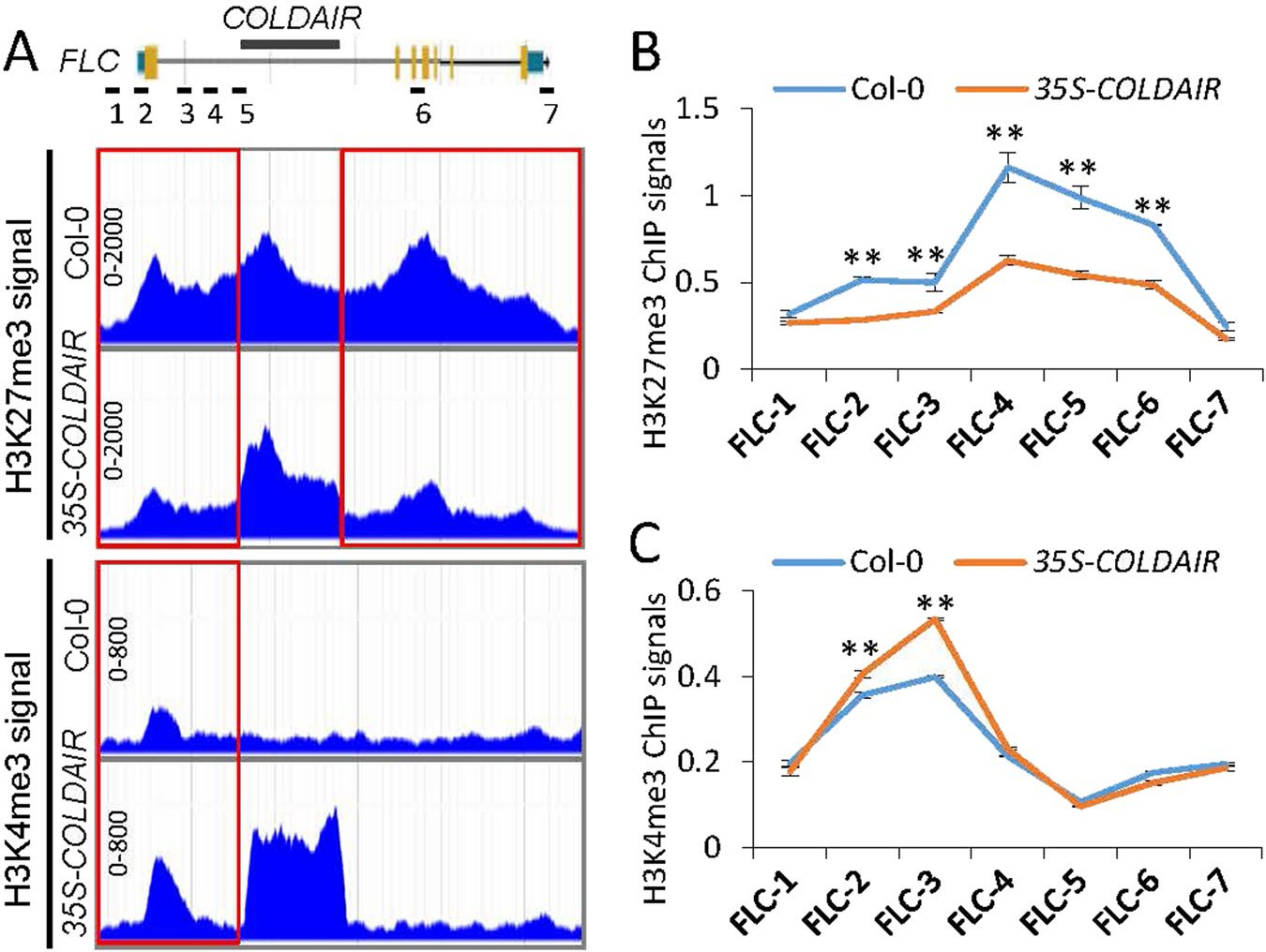
The overexpressed *COLDAIR* suppresses H3K27me3 levels and enhances H3K4me3 levels at the *FLC* locus. **(A)** Snapshots of H3K27me3 and H3K4me3 ChIP-seq signals at the *FLC* genomic locus in the wild type and the *35S-COLDAIR* #3 transgenic line. A schematic representation of the *FLC* genomic locus is at the top. Exons, introns, untranslated regions are indicated by yellow boxes, grey lines, and blue boxes, respectively. The *COLDAIR* locus is labelled. **(B,C)** ChIP-qPCR analysis of H3K27me3 (**B**) and H3K4me3 (**C**) levels at indicated *FLC* regions in the wild type and the *35S-COLDAIR* #3 transgenic line. H3K27me3 and H3K4me3 levels were normalized to those of internal reference genes *STM* and *ACT7*, respectively. Error bars are the SD of three replicates. Asterisks indicate that the enrichment of H3K27me3 is significantly reduced or the enrichment of H3K4me3 is significantly increased in the *35S-COLDAIR* transgenic plants as compared to the Col-0 control at indicated loci (Student’s t test; **p < 0.01).

In *Arabidopsis*, *FLC* chromatin is marked by both H3K27me3, H3K4me3, and H3K36me3^21,48,49^. We carried out chromatin immunoprecipitation followed by deep sequencing (ChIP-seq) to determine whether the exogenously overexpressed *COLDAIR* affects *FLC* expression by regulating H3K27me3 and H3K4me3. Consistent with previous studies^48,49^, our ChIP-seq results indicated that, in the wild type, H3K27me3 was enriched throughout the full length of the *FLC* genomic region and H3K4me3 was specifically enriched at the region shortly after the transcription start site (Fig. 5A). In the *35S-COLDAIR* transgenic line, the H3K27me3 level was significantly lower than in the wild type, whereas the H3K4me3 level was significantly higher than in the wild type (Fig. 5A). The aberrant H3K27me3 and H3K4me3 signals of the *COLDAIR* region in the *35S-COLDAIR* transgenic line were most likely due to the presence of the exogenous *COLDAIR* in the transgenic line, which could not be distinguished from the endogenous *COLDAIR* region by ChIP-seq.

To further validate the effect of the exogenous *COLDAIR* on H3K27me3 and H3K4me3, we performed ChIP followed by PCR (ChIP-PCR) in the wild-type Col-0 control and the *35S-COLDAIR* transgenic line. In particular, we assessed the H3K27me3 and H3K4me3 levels of the endogenous *COLDAIR* region by using a pair of primers that can specifically amplify the fragment (the 5^th^ fragment) covering the edge of the endogenous *COLDAIR* region. The results indicated that the H3K27me3 level of the full-length *FLC* was lower in the *35S-COLDAIR* transgenic line than in Col-0 even in the endogenous *COLDAIR* region (Fig. 5B). The H3K4me3 ChIP-PCR experiment also confirmed the increase of H3K4me3 at the region shortly after the transcription start site of *FLC* in the *35S-COLDAIR* transgenic line (Fig. 5C). Moreover, the H3K4me3 enrichment in the endogenous *COLDAIR* region (the 5^th^ fragment) of the *35S-COLDAIR* transgenic line was not detected by ChIP-PCR (Fig. 5C), confirming that the aberrant H3K27me3 and H3K4me3 signals identified by ChIP-seq in the *COLDAIR* region of the *35S-COLDAIR* transgenic line was from the exogenous *COLDAIR* rather than from the endogenous *COLDAIR*. These results therefore demonstrate that the exogenous *COLDAIR* promotes *FLC* expression by reducing H3K27me3 levels and increasing H3K4me3 levels.

### CLF and ATX1 are involved in the regulation of *FLC* expression by exogenous *COLDAIR*

**Figure 6:**
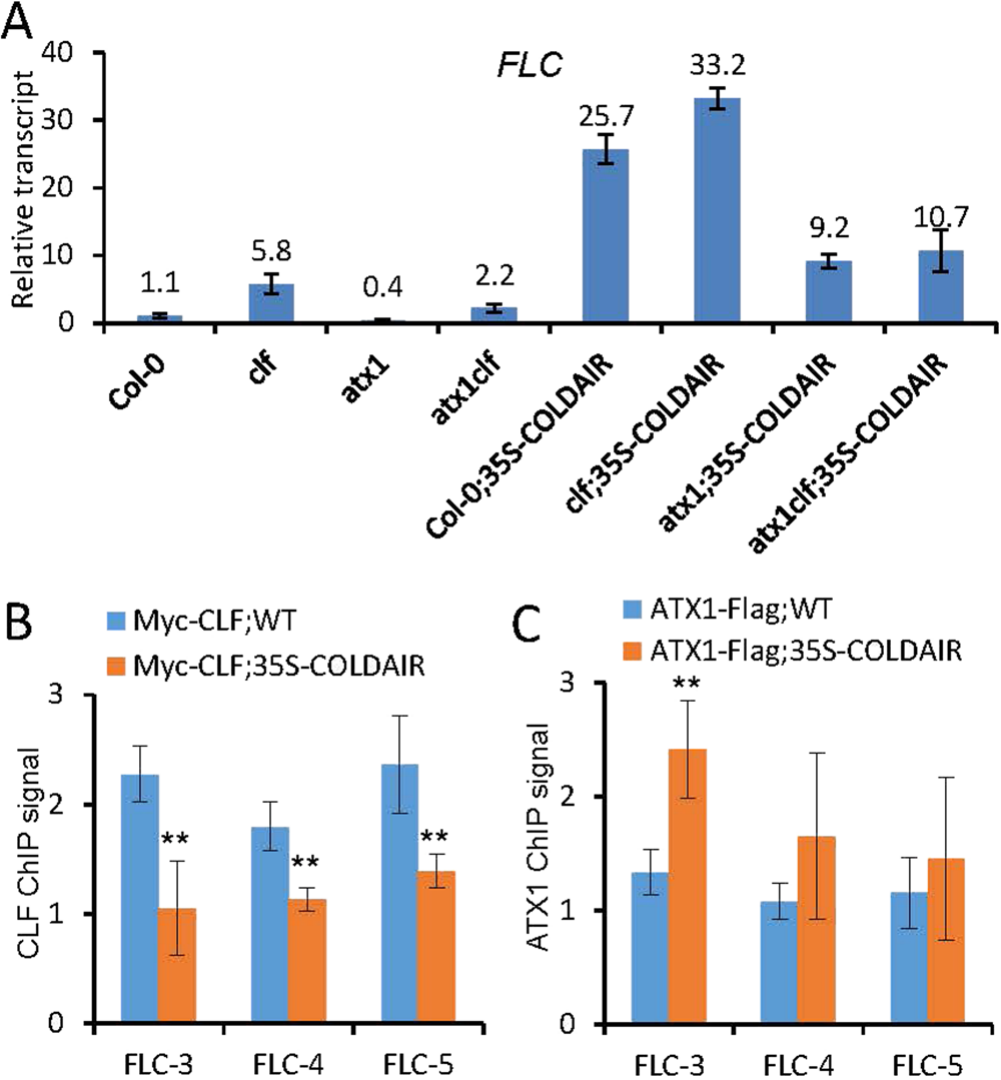
The overexpressed *COLDAIR* affects the occupancy of CLF and ATX1 on the *FLC* locus. **(A)** The effect of *clf* and *atx1* mutations on the activation of *FLC* by *COLDAIR* overexpression. The *FLC* transcript levels were determined by RT-qPCR in the indicated genotypes. The *clf* and *atx1* mutations were introduced into the *35S-COLDAIR* #3 transgenic line by genetic crossing. Error bars are the SD of three replicates. **(B,C)** ChIP-qPCR analysis of tagged CLF (**B**) and ATX1 (**C**) levels at indicated *FLC* regions in the wild type and the *35S-COLDAIR* #3 transgenic line. The overexpressed *COLDAIR* was introduced into *Myc-CLF* and *ATX1-flag* transgenic plants by genetic crossing. The ChIP-qPCR signal on *ACT7* was used as a control for normalization. Error bars are the SD of at least two biological replicates.

CLF is a major histone H3K27 trimethyltransferase in the Arabidopsis PcG complex^24–26^. In the *clf* mutant, the H3K27me3 level of *FLC* is reduced, which results in an increase of *FLC* expression^23^. To investigate whether the PcG complex is related to the increase of *FLC* expression, we crossed the *35S-COLDAIR* line with the *clf* mutant to determine the effect of *clf* on *FLC* expression. Our RT-qPCR result showed that the transcript level of *35S-COLDAIR* is not significantly affected in the *clf* mutant compared to the wild type (Fig. S4). In the wild-type background, the *clf* mutation significantly (Student’s t test; p < 0.01) enhanced *FLC* expression (Fig. 6A). In the *35S-COLDAIR* line, however, the *FLC* expression level was markedly higher than in the wild type, and the *clf* mutation only slightly enhanced *FLC* expression (Fig. 6A). Considering that CLF is a major histone H3K27 trimethyltransferase in the PcG complex, we predicted that the overexpressed *COLDAIR* may promote *FLC* expression at least partially through suppressing the function of the PcG complex in H3K27me3.

Trithorax Group complexes (TrxG) are responsible for establishment of H3K4me3 at the *FLC* chromatin and thereby facilitate *FLC* expression^27^. ATX1 is a major histone H3K4 methyltransferase subunit of the TrxG complex^50,51^. In the *atx1* mutant, the *FLC* expression was reduced, which led to an early-flowering phenotype^27^. To investigate whether the TrxG complex is involved in the regulation of *FLC* expression by the exogenous *COLDAIR*, we crossed the *35S-COLDAIR* line with the *atx1* mutant and determined how the *atx1* mutation affects *FLC* expression in the *35S-COLDAIR* line. The transcript level of *COLDAIR* in the *35S-COLDAIR* line is comparable between the wild-type and *atx1* mutant backgrounds as determined by RT-qPCR (Fig. S4). Consistent with the previous study^27^, our RT-qPCR experiment showed that, the *atx1* mutation significantly reduced *FLC* expression in the wild-type background (Fig. 6A). *FLC* expression was reduced by the *atx1* mutation in the *35S-COLDAIR* line as well as in the wild type even though the basic *FLC* expression level was significantly higher in the *35S-COLDAIR* line than in the wild type (Fig. 6A), suggesting that the increase of *FLC* expression caused by the *COLDAIR* overexpression is partially dependent on ATX1. Given that ATX1 is a major histone H3K4 methyltransferase in the TrxG complex, the results indicate that *FLC* expression depends, at least partially, on the TrxG complex.

To further investigate how the histone H3K27 methyltransferase CLF and the histone H3K4 methyltransferase ATX1 are coordinated to facilitate *FLC* expression in the *35S-COLDAIR* lines, we generated a *clf/atx1* double mutant in the *35S-COLDAIR* and wild-type backgrounds by genetic crossing. RT-qPCR indicated that the transcript level of *COLDAIR* in the *35S-COLDAIR* line is not significantly affected in the *clf/atx1* mutant relative to the wild type (Fig. S4).

In the wild type background, the *FLC* expression level was reduced in the *atx1* mutant, but the reduction of *FLC* expression was partially restored by the *clf* mutation in the *clf/atx1* double mutant (Fig. 6A), suggesting that CLF and ATX1 cooperate to regulate *FLC* expression. In the *35S-COLDAIR* line, however, the reduction of *FLC* expression caused by the *atx1* mutation failed to be restored by the *clf* mutation (Fig. 6A). As indicated above (Fig. 6A), the *clf* mutation also had a very weak effect on *FLC* expression in the *35S-COLDAIR* line even when ATX1 was not mutated. These results suggest that the promotion of *FLC* expression by the *COLDAIR* overexpression is related to CLF and ATX1.

### *COLDAIR* suppresses CLF occupancy and increases ATX1 occupancy on *FLC*

Both CLF and ATX1 were previously shown to directly interact with the *FLC* chromatin in order to regulate transcription^23,27^. We performed ChIP-qPCR using Myc-tagged *CLF* and Flag-tagged *ATX1* transgenic plants to determine whether the overexpressed *COLDAIR* affects the occupancy of CLF and ATX1 at the *FLC* chromatin loci. We found that, in the *35S-COLDAIR* plants, the enrichment of CLF was significantly lower than in the wild type (Fig. 6B), whereas the enrichment of ATX1 was significantly higher than in the wild type (Fig. 6C). These data suggest that the overexpressed *COLDAIR* somehow modulates the occupancy of CLF and ATX1 on the *FLC* chromatin and thereby regulates H3K27me3 and H3K4me3, respectively.

### Activation of host genes by intronic RNAs is conserved in MIKC MADS-box genes

In Arabidopsis, there are 107 genes encoding MADS-box proteins, which are involved in various development processes^52^. Based on the homology of the conserved MADS box, 39 MADS-box proteins including FLC were classified as MIKC MADS-box proteins^52^. Many MIKC MADS-box genes are enriched with H3K27me3 as determined by whole-genome ChIP-seq analyses^48,53^. Because overexpression of the first intron of *FLC* enhances *FLC* expression through repression of H3K27me3, we asked whether the function of intronic lncRNAs is conserved in the other MIKC MADS-box genes.

**Figure 7:**
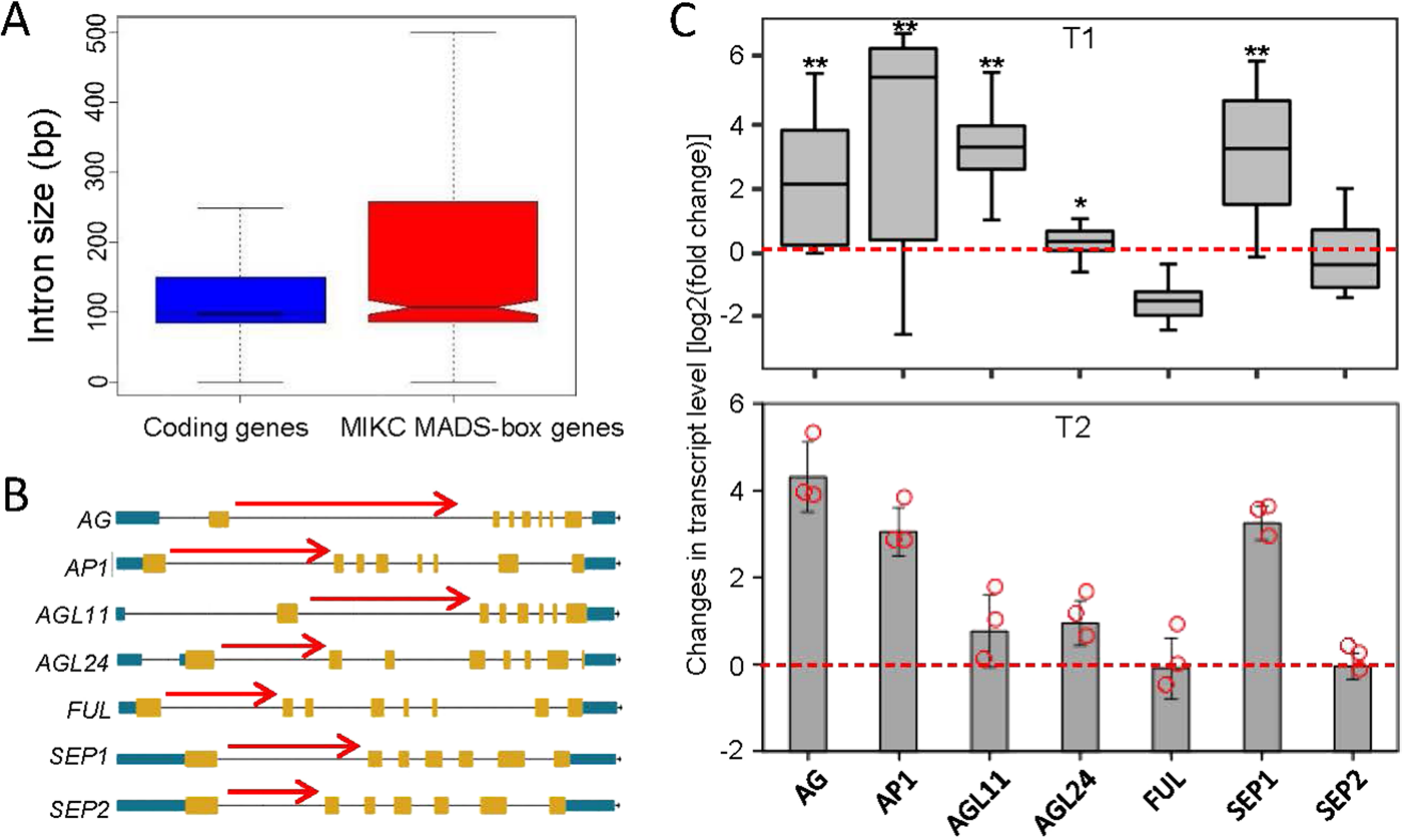
The function of intronic RNAs in activating gene expression is conserved in MIKC MADS-box genes. **(A)** Box plots showing the intron sizes of MADS-box genes and all protein-coding genes in Arabidopsis. **(B)** Schematic representation of seven MADS-box genes and the intronic RNAs (red arrows) overexpressed in transgenic plants. **(C)** The effect of overexpressed intronic RNAs on the expression of corresponding MADS-box genes. The effects in 15 randomly selected intronic RNA overexpressed T1 transgenic plants and in representative T2 transgenic lines are shown in the top and bottom panels, respectively. In the bottom panel, each cycle denotes an individual T2 transgenic line. The expression of the MADS-box genes was determined by RT-qPCR.

We assessed the intron length of MIKC MADS-box genes and found that the MIKC MADS-box genes have larger intron sizes than the other protein-coding genes in Arabidopsis (Fig. 7A). Therefore, we predicted that overexpression of intronic lncRNAs from the other MIKC MADS-box genes may also enhance the expression of their corresponding host genes. To validate the function of intronic lncRNAs in the other MIKC MADS-box genes, we selected seven additional MIKC MADS-box genes including *AGAMOUS* (*AG*), *AP1*, *AGAMOUS-LIKE 11* (*AGL11*), *AGAMOUS-LIKE 24* (*AGL24*), *SEPALLATA 1* (*SEP1*), *FRUITFULL* (*FUL*), and *SEPALLATA 2* (*SEP2*) for analysis (Fig. 7B). By overexpressing the longest intronic RNAs of the seven MIKC MADS-box genes (Fig. 7B), we found that the overexpressed intronic RNAs were capable of activating the expression of *AG*, *AP1*, *AGL11*, *AGL24*, and *SEP1* but not the expression of *FUL* and *SEP2* in their corresponding T1 transgenic plants (Fig. 7C). The effect of these intronic RNAs on the expression of *AG*, *AP1*, *AGL11*, *AGL24*, and *SEP1* can be inherited in the transgenic T2 plants (Fig. 7C). These results demonstrate that enhancement of the expression of host genes by overexpressing their corresponding intronic RNAs is conserved for MIKC MADS-box genes.

## Discussion

In Arabidopsis, the endogenous *COLDAIR* was previously shown to associate with the major H3K27 methyltransferase CLF and thereby enhance H3K27me3 at the late stage of vernalization^12^. Unlike the previous study, the results in the current study strongly suggests that the exogenously overexpressed *COLDAIR* suppresses H3K27me3 at the *FLC* chromatin, indicating that the exogenous *COLDAIR* shows an antagonistic effect on H3K27me3 compared to the endogenous *COLDAIR*. We predict that the exogenously overexpressed *COLDAIR* may act as an inhibitor or a decoy for CLF and thereby make the cis-acting endogenous *COLDAIR* inaccessible to CLF for H3K27me3 establishment. Alternatively, it may also be possible that antagonistic effects of *COLDAIR* on H3K27me3 occur during different biological processes. The promotion of H3K27me3 by *COLDAIR* occurs during vernalization, whereas the suppression of H3K27me3 by *COLDAIR* may occur in the other biological processes such as early embryo development when *FLC* expression is reactivated.

In the exogenously overexpressed *COLDAIR* line, H3K27me3 is reduced, and H3K4me3 is increased (Fig. 5A,B). The dual effect on H3K27me3 and H3K4me3 is also observed in the mutant defective in the histone H3K27 tri-methyltransferase CLF^23^. Therefore, it is possible that H3K27me3 may be primarily reduced by the exogenous *COLDAIR* and the reduction of H3K27me3 may subsequently promote the establishment of H3K4me3. In humans, the lncRNA *HOTTIP* transcribed from the 5′ end of the *HOXA* locus directly associates with WDR5, a subunit of the H3K4 trimethyltransferase complex COMPASS and thereby promotes *HOXA* expression by increasing H3K4me3^8^. Components of the COMPASS complex in Arabidopsis, which are conserved with those in mammals, have been demonstrated to bind to *FLC* and its homologs and are required for promoting H3K4me3^54^. In Arabidopsis, an antisense lncRNA was shown to interact with WDR5a to recruit the COMPASS complex to *MAF4*, a flowering repressor gene closely related to *FLC*, leading to an increase in H3K4me3^55^. Our results indicate that the overexpressed intronic RNA derived from the intron 1 of *FLC* increases *FLC* expression and H3K4me3 by reinforcing the occupancy of ATX1 on the *FLC* chromatin. Because ATX1 is a major catalytic subunit of the Arabidopsis COMPASS complex, we propose that the intronic RNA may also directly associate with the COMPASS subunit WDR5a and thereby recruit the COMPASS complex to the *FLC* chromatin for H3K4me3. However, we cannot exclude the possibility that the mechanisms other than histone H3K4 and H3K27 methylation may also be involved in the promotion of *FLC* expression by the overexpressed *COLDAIR*. Considering that the coupled changes in H3K27me3 and H3ac were recently observed^56^, we predict that the histone acetylation may also be necessary for the promotion of *FLC* expression by the overexpressed *COLDAIR*.

By overexpressing intronic RNAs derived from randomly selected MIKC MADS-box genes, we demonstrated that the activation of gene expression by overexpressed intronic RNAs is conserved in the MIKC MADS-box genes *AG*, *AP1*, *AGL11*, *AGL24*, and *SEP1*, but not in *FUL* and *SEP2* (Fig. 7B,C). Of note, *FLC* expression was enhanced in only a subset of the *35S-COLDAIR* lines (Fig. 1A–D), suggesting that some uncharacterized mechanisms may be responsible for counteracting the activation of the host genes by overexpressed intronic RNAs. Our results showed that *FLC* expression is enhanced in the *dcl2/3/4* mutant (Fig. 6A), which is consistent with the previous finding that DCL3 promotes flowering by reducing *FLC* expression^57,58^. Given that a large number of small RNAs are generated from the *COLDAIR* locus in the *35S-COLDAIR* line, we predict that these small RNAs may be responsible for the reduction of *FLC* expression at either transcriptional or posttranscriptional levels, thereby counteracting the promotion of *FLC* expression by the overexpressed *COLDAIR*. We suspect that the function of overexpressed intronic RNAs in activating gene expression by reducing H3K27me3 may be conserved in H3K27me3-enriched genes in Arabidopsis. Because H3K27me3 occurs not only in MADS-box genes but also in thousands of other genes^48,53^, the function of overexpressed intronic RNAs may be much more common than we expected in H3K27me3-enriched genes. Considering that stable intronic RNAs have been extensively identified in eukaryotes^30,32,33^, we predict that these endogenous intronic RNAs may also play important roles in the regulation of gene expression through the same mechanism.

Repressed *FLC* expression needs to be reactivated during the early embryo development and therefore ensures the requirement for vernalization-induced H3K27me3 in every generation. The transcriptional activators LEAFY COTYLEDON 1 (LEC1), LEAFY COTYLEDON 2 (LEC2) and FUSCA 3 (FUS3) and the histone H3K27 demethylase EARLY FLOWERING 6 (ELF6) are required for the reactivation of *FLC* by reducing H3K27me3 levels^59–61^. Because the transcriptional activators required for *FLC* expression are exclusively expressed in the embryo^59,60^, it is unknown how *FLC* expression is maintained after the embryo-to-seedling transition. Moreover, loss of REF6 only partially affects H3K27 demethylation at *FLC* chromatin during reproductive development^61^, suggesting that an REF6-independent mechanism must be required for reducing H3K27me3 levels. The results from the current study indicate that the overexpressed intronic RNA from the intron 1 of *FLC* is sufficient for reducing H3K27me3 levels. We suspect that the embryo-specific transcriptional activators may be responsible for initiating the reactivation of *FLC* expression by reducing H3K27me3 during the early embryo development. With the reactivation of *FLC* expression, the intronic RNA excised from the intron 1 of *FLC* pre-mRNA may gradually accumulate, and the accumulated intronic RNA would further suppress H3K27me3 and increase H3K4me3, resulting in a full activation of *FLC* expression after the embryo-to-seedling transition. Based on all of these results, we suspect that overexpressed intronic RNAs may activate gene expression by counteracting H3K27me3 and/or by promoting H3K4me3 during important biological processes.

## Materials and Methods

### Plant materials and plasmid construction

The Arabidopsis materials were all in the Columbia (Col-0) background and were grown on Murashige and Skoog (MS) medium at 22 °C with 16-h light/8-h dark (long-day conditions). T-DNA mutants *flc-8* (Salk_072590C), *clf* (Salk_088542C), and *atx1* (SAIL_409_A10) were obtained from the Arabidopsis Biological Resource Center. The *dcl2/3/4* triple mutant was previously reported^62^. To construct the intronic RNA overexpressed transgenic plants, full-length or truncated intron sequences of target genes were inserted downstream of the constitutive *CaMV 35S* promoter in the binary vector *pCambia1300* via ClonExpress II One Step Cloning Kit (Vazyme Biotech). The constructs were then transformed into wild-type (Col-0) plants by Agrobacteria infection. T1 transgenic plants were grown on MS medium plates supplemented with 25 mg/L hygromycin to screen the resistant seedlings for further study. For *ATX1-Flag* construction, an 8349-bp *ATX1* genomic fragment (including a 1618-bp region upstream of the translation start codon and a 6731-bp genic region without the translation stop codon) was fused in frame with three copies of Flag (3 × Flag) tag, and was cloned into the binary vector *pCAMBIA1305*. The *ATX1-Flag* and *Myc-CLF* transgenic plants were crossed to the *35S-COLDAIR* line and the plants harboring both the transgene and the overexpressed *COLDAIR* were selected from the F2 segregation group. The DNA primers used for construction are listed in Data set 2.

### RNA extraction and RNA level analysis

RNA was extracted from 14-day-old plants as described previously^63^. The first strand of cDNA was prepared with the PrimeScript RT Reagent Kit (TAKARA, RR037A). Quantitative PCR was performed on an ABI 7500 fast Real time PCR instrument with KAPA SYBR FAST Universal reaction regent, and the results were quantified by reference to a standard curve for each primer pair with at least two repeats. For assessment of noncoding RNA expression, sequence-specific primers were used for reverse transcription. For RNase-treated RT-qPCR, 10 µg of total RNA was digested with RNase III (NEB, M0245S) or RNase one (Promega, M4261) according to the instruction manual. After chloroform purification, 1 µg of RNA was treated with DNase I to remove genomic DNA, and the RNA was then subjected to RT-PCR. Oligonucleotides used in this study are indicated in Data set 2.

### Transcriptome sequencing and data analysis

Total RNA was extracted from 12-day-old seedlings, and mRNA was isolated from the total RNA using poly-T oligo-attached magnetic beads. The Illumina ScriptSeq Complete Kit (Plant) was used for library construction. Small RNAs of 18–60 nt were gel-purified and subjected to library construction. Library construction and deep sequencing were performed by Vazyme Biotech (Nanjing, China). For analysis of transcriptome sequencing data, clean reads were mapped to the TAIR10 Arabidopsis genome by TopHat v2.1.0^64^, allowing up to two mismatches. The differentially expressed genes were calculated by cuffdiff v2.0.1^65^. For analysis of small RNA deep sequencing data, 18 to 30-nt clean reads were mapped to the TAIR10 Arabidopsis genome using Bowtie^66^, and only perfectly matched reads were retained for further analysis.

### ChIP assay

The ChIP experiment was performed according to a previous report with minor modification^67^. A 2-g quantity of 2-week-old seedlings was cross-linked in 1% formaldehyde for 20 min after ground. The cross-linking reaction was stopped by adding glycine to 125 mM and was incubated for 5 min. The chromatin was isolated with Nuclear Extraction Buffer [20 mM Tris-HCl (pH 7.5), 20 mM KCl, 2 mM EDTA (pH 8.0), 2.5 mM MgCl_2_, 25% glycerol, 250 mM sucrose, 5 mM DTT, 1 mM PMSF, and 1 × protease inhibitor mixture (Roche)]. After they were washed twice on two layers of Miracloth with Nuclear Extraction Buffer, and 3–5 times with Nuclear Resuspension Buffer [20 mM Tris-HCl (pH 7.5), 2.5 mM MgCl_2_, 25% glycerol, 0.2% Triton X-100], the nuclear pellets were resuspended in 800 μL of Nuclei Lysis Buffer [20 mM Tris-HCl (pH 8.0), 2 mM EDTA, 0.2% NP-40, 1 mM PMSF, and 1 × protease inhibitor mixture). The chromatin of each sample was sonicated by a Bioruptor Plus device at a high power level for 28–33 cycles (30 sec ON and 30 sec OFF for each cycle). A 800-μL volume of sonicated chromatin diluted with ChIP dilution buffer (1 mM PSMF, 2 mM EDTA pH 8.0, 20 mM Tris–HCl pH 8.0, 200 mM NaCl, and 1 × protease inhibitor mixture) to 1.5 mL was used for immunoprecipitation. Dynabeads Proein A (Thermo, 10001D) were used for conjugating the anti-bodies. The anti-bodies used in this study were anti-H3 (Abcam, ab1791), anti-H3K4me3 (Millipore, 07–473), anti-H3K27me3 (Millipore, 07–449). The conjugated antibodies were independently mixed with the chromatin by rotation at 4 °C overnight. Beads were washed five times with Wash Buffer (150 mM NaCl, 20 mM Tris-HCl pH 8.0, 2 mM EDTA pH 8.0, 0.1% Triton X-100, 1 mM PMSF). Finally, beads were washed twice for 5 min each time with TE buffer (10 mM Tris, pH 7.5, 1 mM EDTA). After they were washed, the immuno-complexes was eluted from the beads and reverse cross-linked by NaCl and 20 ug Proteinase K (Sigma, P4850). Phenol/Chloroform/Isoamyl alcohol was used to extract the ChIP-DNA and followed with qPCR or sequencing. Deep sequencing was performed by Novogene (Beijing, China).

For ChIP analysis of Myc-CLF and ATX1-Flag, nuclei were isolated as described above. After washing with Honda buffer [20 mM HEPES (pH 7.4), 0.44 M Sucrose, 1.25% (wt/vol) Ficoll, 2.5% (wt/vol) Dextran T40, 10 mM MgCl_2_, 0.5% Triton X-100, 5 mM DTT, 1 × protease inhibitor mixture (Roche)], the nuclei were resuspended with IP Binding Buffer (50 mM Tris-HCl pH 8, 150 mM NaCl, 5 mM MgCl_2_, 5% glycerol, 0.1% NP-40, 1 mM PMSF, and protease inhibitor cocktail). The chromatin was then sheared by sonication and centrifuged at 5000 rpm for 10 min, and the supernatant was immunoprecipitated with Anti-c-Myc Agarose Affinity Gel (Sigma, A7470) or Anti-FLAG M2 Magnetic Beads (Sigma, M8823). The beads were then washed twice with IP Binding Buffer containing 500 mM NaCl. The sample was then treated as above for qPCR.

### Stranded mRNA sequencing

Total RNA was extracted from 14-day-old seedlings are subjected to the Vazyme VAHTS Total RNA-seq Library Prep Kit for deep sequencing (Vazyme, NR603). RNA were digested with RNase H and DNase I and purified with VAHTS RNA Clean Beads (Vazyme, N412). Ribosome-depleted RNA was used as a template for synthesis of both strands of cDNA. Double stranded cDNA was purified with VAHTS DNA Clean Beads (Vazyme, N411). The dA-tailing and adapter ligation were performed using VAHTS RNA Adapters Set 1 - Set 2 (Vazyme, N803, N804). 1 × VAHTS DNA Clean Beads (Vazyme, N411) was used for purification and size selection of adapter-ligated DNA followed by amplification. Agilent DNA 1000 chip (Agilent, 5067-1504) was used for determination of library qualitydetermination.

## Supplementary information

Supplementary information.

Supplementary information2.

## Data Availability

The raw data of RNA-seq, small RNA-seq, and ChIP-seq were deposited in the Gene Expression Omnibus (GEO) database (accession number: GSE140140).
