# Publisher Correction: Exogenously overexpressed intronic long noncoding RNAs activate host gene expression by affecting histone modification in Arabidopsis

**DOI:** 10.1038/s41598-020-79079-3

**Published:** 2020-12-23

**Authors:** Zhang-Wei Liu, Nan Zhao, Yin-Na Su, Shan-Shan Chen, Xin-Jian He

**Affiliations:** 1 National Institute of Biological Sciences, Beijing, 102206 China; 2 Tsinghua Institute of Multidisciplinary Biomedical Research, Tsinghua University, 10084 Beijing, China

Correction to: *Scientific Reports* 10.1038/s41598-020-59697-7, published online 20 February 2020

The original version of this Article contains an error where two supplementary files containing raw data, which were included with the initial submission, were omitted. These files appear below.

## Supplementary Information

Supplementary Data set 1.

Supplementary Data set 2.

