# Supplementary material for: Exogenously overexpressed intronic long noncoding RNAs activate host gene expression by affecting histone modification in Arabidopsis
Source: Sci Rep. 2020 Feb 20;10:3094. doi: 10.1038/s41598-020-59697-7 (PMC7033118; doi:10.1038/s41598-020-59697-7)
Supplement: Supplementary file 2 — Supplementary information2. [file 41598_2020_59697_MOESM2_ESM.pdf]

Figure S1

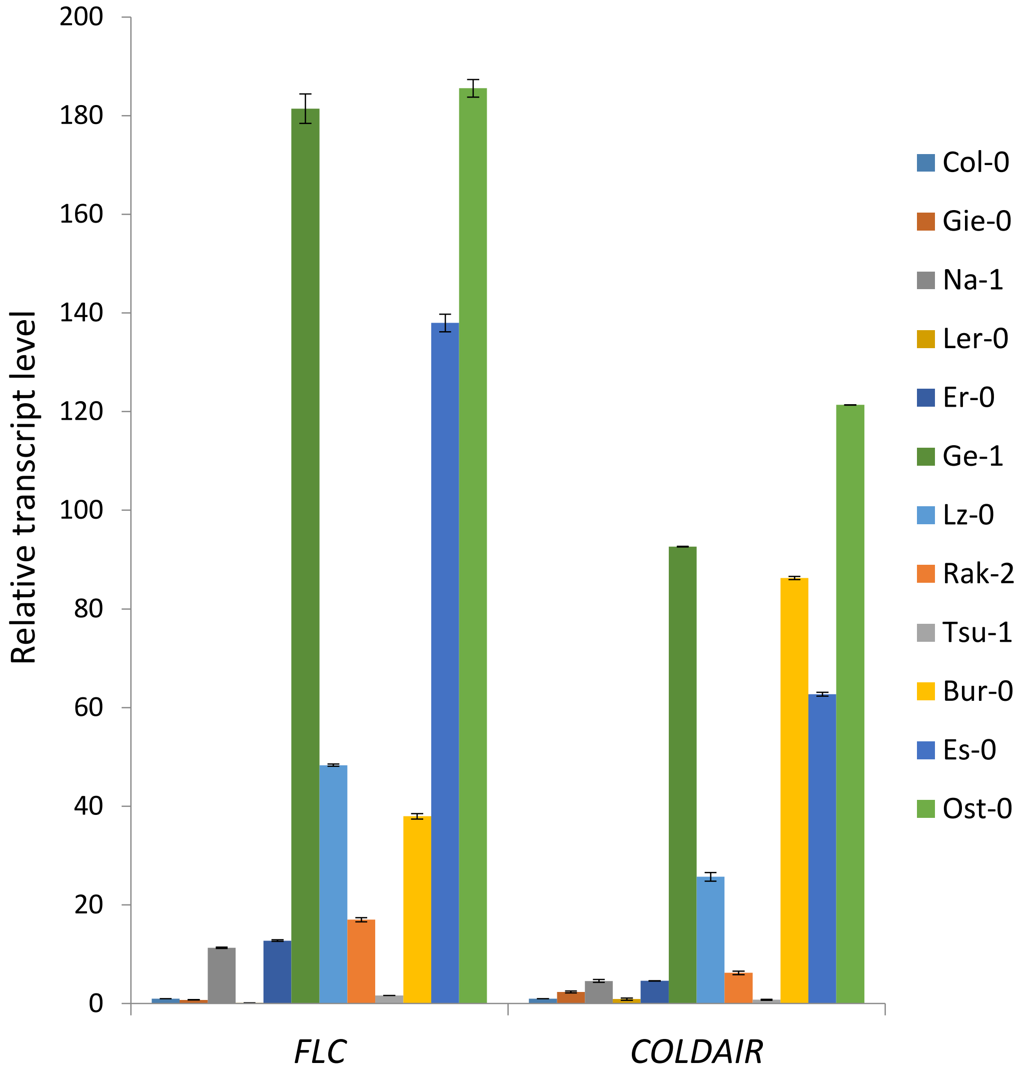

Figure S1. The transcript levels of *FLC* and *COLDAIR* in different ecotypes of Arabidopsis. The transcript levels of *FLC* and *COLDAIR* were determined by RT-qPCR in indicated ecotypes of Arabidopsis. The transcript level of *PP2A* was used as an internal control. Error bars are SD of three replicates.

Figure S2

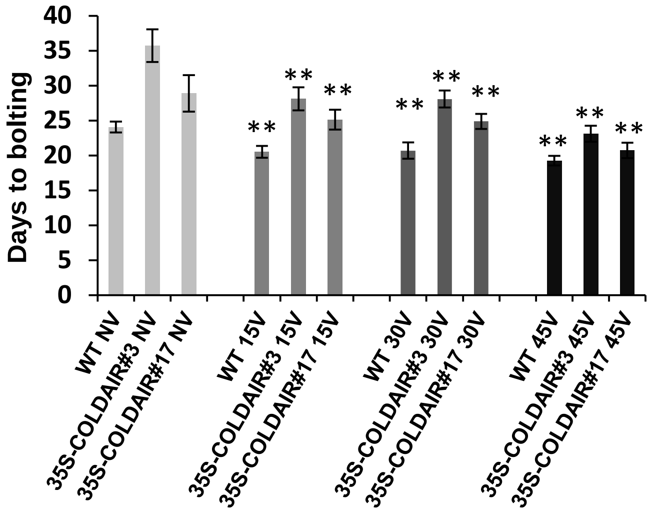

Figure S2. The effect of vernalization on flowering time in wild-type and *COLDAIR* overexpressed plants. Flowering time was quantified by days to flower. NV represents non-vernalized plants; V15, V30, and V45 represent plants that were vernalized for 15, 30, and 45 days, respectively. Asterisks indicate statistically significant differences between non-vernalization and 15, 30, or 45-day vernalization treatments (Student's t test; **P<0.01).

Figure S3

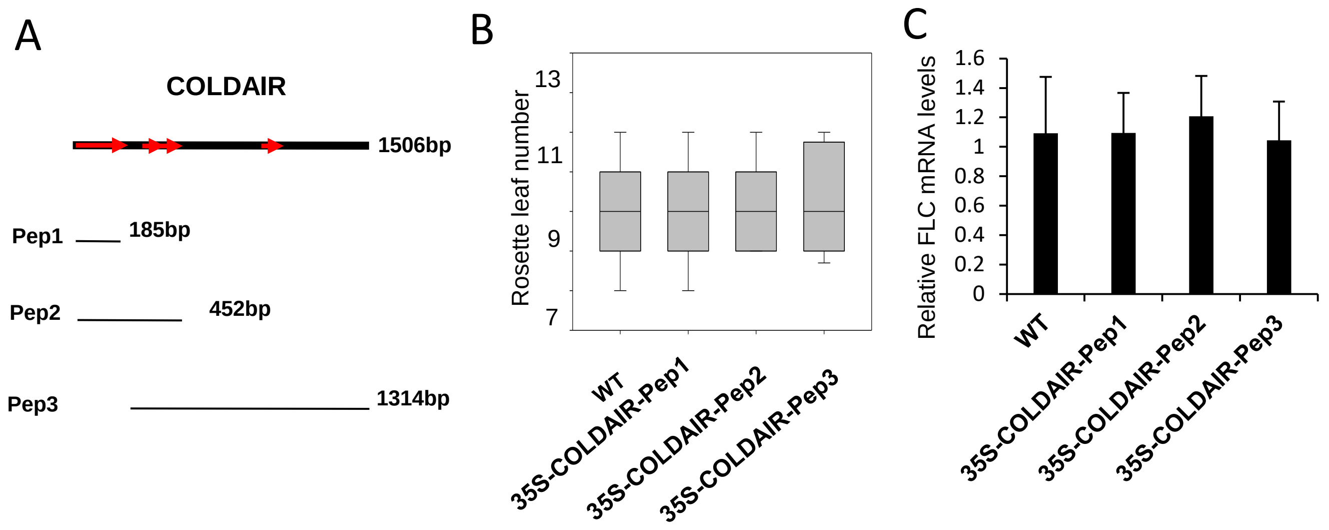

Figure S3. The function of the over-expressed *COLDAIR* in activation of *FLC* is independent of the production of small peptides. (A) Schematic representation of full-length and truncated transcripts of *COLDAIR* used in over-expressed transgenic plants. Red arrows represent the putative small open reading frames predicted by NCBI ORF Finder (https://www.ncbi.nlm.nih.gov/orffinder/). **(B)** Box plots showing rosette leaf numbers of truncated *COLDAIR* over-expressed T1 transgenic plants in long days. Thirty-six independent T1 transgenic plants were scored for each version of truncated *COLDAIR*. **(C)** *FLC* mRNA levels in wild-type plants and truncated *COLDAIR* over-expressed T2 transgenic plants. At least 10 individual T2 transgenic lines were used for RT-qPCR. The expression of the *PP2A* gene was determined as an internal control. Error bars are SD of three biological replicates.

Figure S4

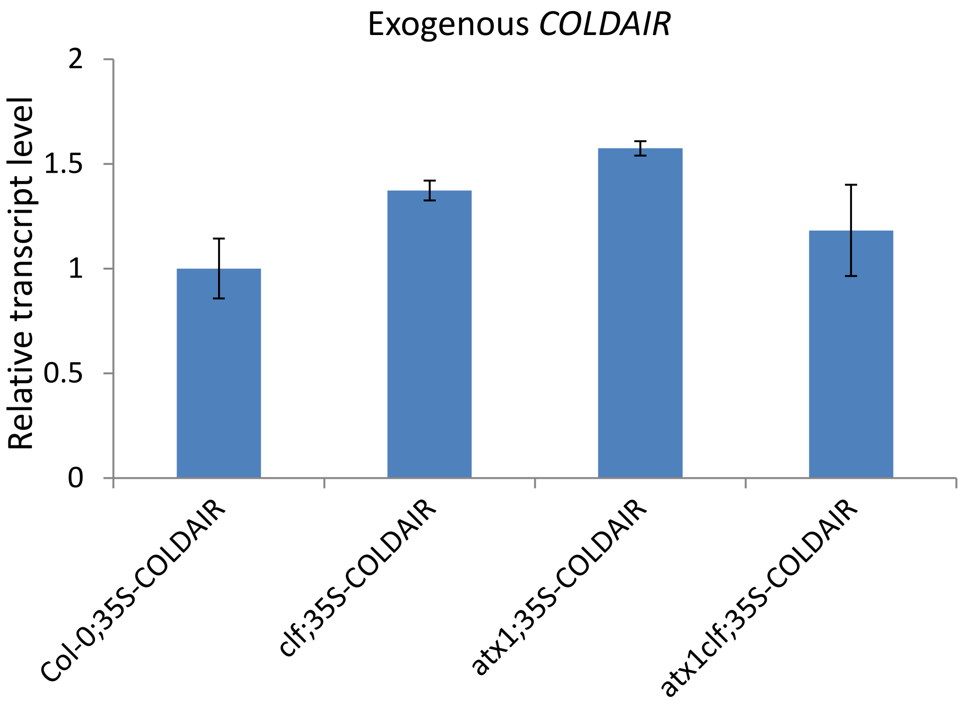

Figure S4. The transcript level of exogenous *COLDAIR* in the *35S-COLDAIR* transgenic lines of different backgrounds. The transcript level of exogenous *COLDAIR* was determined by RT-qPCR in the wild type, *clf*, *atx1*, and *clf/atx1* mutant backgrounds. The transcript level of *PP2A* was used as an internal control. Error bars are SD of three replicates.
